# RNA N6-Methyladenosine Regulator-Mediated Methylation Modifications Pattern and Immune Infiltration Features in Glioblastoma

**DOI:** 10.3389/fonc.2021.632934

**Published:** 2021-02-25

**Authors:** Yimin Pan, Kai Xiao, Yue Li, Yuzhe Li, Qing Liu

**Affiliations:** Department of Neurosurgery in Xiangya Hospital, Central South University, Changsha, China

**Keywords:** glioblastoma, m^6^A, immune infiltration, immunotherapy, prognosis

## Abstract

Glioblastoma (GBM) is a group of intracranial neoplasms with intra-tumoral heterogeneity. RNA N6-methyladenosine (m^6^A) methylation modification reportedly plays roles in immune response. The relationship between the m^6^A modification pattern and immune cell infiltration in GBM remains unknown. Utilizing expression data of GBM patients, we thoroughly explored the potential m^6^A modification pattern and m^6^A-related signatures based on 21 regulators. Thereafter, the m^6^A methylation modification-based prognostic assessment pipeline (MPAP) was constructed to quantitatively assess GBM patients’ clinical prognosis combining the Robustness and LASSO regression. Single-sample gene-set enrichment analysis (ssGSEA) was used to estimate the specific immune cell infiltration level. We identified two diverse clusters with diverse m^6^A modification characteristics. Based on differentially expressed genes (DEGs) within two clusters, m^6^A-related signatures were identified to establish the MPAP, which can be used to quantitatively forecast the prognosis of GBM patients. In addition, the relationship between 21 m^6^A regulators and specific immune cell infiltration was demonstrated in our study and the m^6^A regulator ELAVL1 was determined to play an important role in the anticancer response to PD-L1 therapy. Our findings indicated the relationship between m^6^A methylation modification patterns and tumor microenvironment immune cell infiltration, through which we could comprehensively understand resistance to multiple therapies in GBM, as well as accomplish precise risk stratification according to m^6^A-related signatures.

## Introduction

Glioblastoma (GBM) is the most common lethal neoplasm of the central nervous system, accounting for approximately half of primary brain tumors and almost 60% of all types of gliomas (1). Even after complete surgical removal combined with adjuvant therapy, for example radiotherapy, chemotherapy, and targeted therapy, its prognosis remains notably poor with an extremely low 5-year survival rate of approximately 5% (1–3). In addition, GBM patients and families suffer a heavy burden due to progressive neurological deficits and decreasing quality of life (4). Despite the killing effect of systemic therapy after complete resection, infiltrating cancer cells can often escape, resulting in tumor recurrence, progression, and even death (5). Recent advances in precision oncology, immunology, and other disciplines have uncovered multiple experimental therapies, such as immunotherapy, gene therapy, and novel drug-delivery technologies, which are emerging as powerful tools to solve the complicated GBM treatment difficulties, including low permeability of the blood-brain barrier, complex tumor signaling pathways, and the absence of specific biomarkers (6). Since multimodality therapy heralds promise in achieving durable and broad anticancer responses, it is urgent to establish a reliable tumor classification and prognosis model for cancer treatment strategy planning.

Represented by immune checkpoint inhibitors (ICIs), Chimeric Antigen Receptor T-Cell immunotherapy (CAR-T), cancer vaccines, and oncolytic viruses, immunotherapy produces sustained killing of cancer cells by activating the patients’ own immune system. Since these immunotherapies reportedly produce durable effects on several cancers, these methods have also been applied to primary intracranial malignancies, including newly diagnosed and recurrent glioblastoma (7, 8). The existence of the blood-brain barrier (BBB) and tumor microenvironment (TME) prevents the immune system’s continuous and effective response on intraparenchymal lesions, which limits the application on CNS tumors, resulting in only a specific subgroup of glioma patients benefitting from this treatment (9, 10). Recent studies regarding the tumor microenvironment have challenged the traditional cognition that tumor tissue is composed of pure tumor cells (11). It holds that the core tumor cells are surrounded by a complex microenvironment, which consists of multiple components, such as newborn blood vessels, multiple cell factors, extracellular matrix (ECM), fibroblasts, and immune cells. Immune cells infiltrating the TME were confirmed to be predictive to patients’ clinical outcomes and have a critical role on the immune response affecting the efficacy of immunotherapy, indicating that identifying the infiltrating pattern of immune cells in TME is of great significance to estimate the prognosis of GBM patients and assess the value of various therapies (12, 13).

Recently, it was reported that the epigenetic modification of RNA has a potential specific dependence with microenvironment infiltrating immune cells, suggesting that elucidating the epigenetic characteristics of GBM can provide a comprehensive basis for immunotherapy (14). Among over 150 RNA modifications, N6-methyladenosine (m^6^A) RNA methylation is the most dominant form of epigenetic regulation, occupying approximately 0.3% of total adenosine residues (15–17). Three types of distinct m^6^A regulatory factors called “writers”, “erasers”, and “readers”, respectively, dynamically regulate the process of RNA translation, degradation, and nuclear export by methyltransferases, demethylases, and binding proteins, separately (18). In total, 21 regulators participate in the m^6^A RNA methylation process, among which RBM15, ZC3H13, METTL3, METTL14, WTAP, and KIAA1429 represent the methyltransferases, while FTO and ALKBH5 catalyze the demethylation process. The remaining regulators, such as YTHDF1/2/3, are a group of RNA-binding proteins identifying specific m^6^A methylation regions to regulate downstream translation processes (19, 20). Although previous studies suggested YTHDF family have the role of enhancing translation, mRNA degradation, simultaneously accelerating translation and mRNA degradation respectively through binding different m^6^A-modified region (21–26), a novel model of YTHDF proteins shows that they bind the same mRNA and co-mediate mRNA degradation and cellular differentiation (27). A growing number of studies suggested that m^6^A regulators participate in multiple biological processes during tumor progression, thus elucidating the relationship between m^6^A regulators and tumor microenvironment infiltrating immune cells can assess GBM patients’ anticancer response to immunotherapy (28–30).

Traditional bulk sequencing provides genetic information at the resolution of individual samples, thus there is a limitation whereby it cannot identify specific cells in the given tissue. Hence, single-cell RNA sequencing (scRNA-seq) emerged as a practical tool to thoroughly distinguish each cell cluster, including immune cells in normal and tumor tissue (31). Due to the expensive sequencing costs, scRNA-seq cannot easily translate into clinical setting, and is primarily used for laboratory research only. In order to efficiently estimate immune cell infiltration level, we applied a relative quantitative algorithm based on single-sample gene-set analysis (ssGSEA), which can utilize traditional bulk expression profile data to determine the relative abundance of 23 immune cells in tumor tissue (32, 33). Additionally, by analyzing the correlation among expression patterns of 21 m^6^A methylation regulators, we established the m^6^A methylation-based prognostic assessment pipeline (MPAP) to calculate GBM patients’ m^6^A modification score (MMS). According to the MMS, we can further predict the clinical outcomes of GBM patients. Using the MPAP, we can determine m^6^A modification patterns in disease tissue by using only conventional bulk transcriptome data, which provides novel perspectives of GBM in an efficient and inexpensive way.

## Materials and Methods

### Patient Selection and Data Preprocessing

From Gliovis (gliovis.bioinfo.cnio.es), a published data visualization web tool for brain tumor expression profile data uploaded on Gene-Expression Omnibus (GEO) and the Cancer Genome Atlas (TCGA) (34), six glioblastoma datasets (Donson *et al*, n = 21; Ducray *et al*, n = 48; Gravendeel *et al*, n = 163; Kamoun *et al*, n = 19; Murat *et al*, n = 84; Rembrandt *et al*, n = 209); (tumor = 495; normal = 49) and corresponding clinical data were obtained for downstream analysis, which was sequenced using Affymetrix expression arrays (HG-U133_Plus_2, HG-U133A, HG_U95Av2, and HuGene-1_0-st). Before acquiring the expression data, the data had undergone robust multi-array average normalization, followed by quantile normalization using R package “affy.” The median of genes with multiple probe sets was selected as the final expression value. To eliminate the batch effect produced not by biological differences but by technical biases, we adopted the “Combat” function in the sva package based on the classical Bayesian algorithm. In addition, somatic datasets for glioblastoma and low-grade glioma were obtained from TCGA and Copy Number Variation (CNV) data was downloaded from the UCSC Xena website (https://xena.ucsc.edu/).

### M^6^A Regulators Clustering

To further explore the regulation mode of m^6^A regulators, we extracted expression profiles of 21 m^6^A regulators from integrated GBM microarray datasets. Eight methylases (METTL3/14, RBM15/15B, WTAP, KIAA1429, CBLL1, ZC3H13), two demethylases (ALKBH5, FTO), and 11 RNA binding proteins (YTHDC1/2, YTHDF1/2/3, IGF2BP1, HNRNPA2B1, HNRNPC, FMR1, LRPPRC, ELAVL1) were included for unsupervised clustering analysis. Thereafter, we utilized the ConsensusClusterPlus package to run an unsupervised consensus clustering one thousand times to divide GBM patients into stable subgroups based on different m^6^A modification patterns (35). The R package of ConsensusClusterPlus was used to classify patients with qualitatively different m^6^A modification patterns based on the expression of 21 m^6^A regulators, and two distinct modification patterns were eventually identified using unsupervised clustering, including 233 cases in pattern A and 262 cases in pattern B. We termed these patterns as m^6^A cluster A-B, respectively. Additionally, we conducted a principle component analysis (PCA) of 21 regulators of GBM expression data to explore different m^6^A modification patterns between tumor and normal tissue, as well as each GBM cluster based on consensus clustering.

### Assessment of Immune Cell Infiltration

To estimate immune cell infiltration level, we applied single-sample gene-set enrichment analysis (ssGSEA) using traditional microarray expression data (36). To identify multiple immune cells using ssGSEA, a specific gene set, including gene expression features of 23 immune cells, was employed (32, 33). We obtained an enrichment score for each sample, representing the relative infiltration level of immune cells, using ssGSEA.

### Gene Set Variation Analysis (GSVA) and GO/KEGG Annotation

We downloaded KEGG pathway gene sets, named C2 collection, from the molecular signature database (MsigDB) (https://www.gsea-msigdb.org/gsea/msigdb) for GSVA inputting.^29^ Next, we performed GSVA using R package “GSVA” on each subgroup to compare relative enrichment level of immune-related KEGG pathways (37). Furthermore, differentially expressed genes (DEGs) among subgroups of distinct m^6^A modification patterns were utilized for GO and KEGG enrichment analysis based on R package “ClusterProfiler”, which uses hypergeometric distribution tests to annotate DEGs (38).

### Differential Expression Analysis

We performed a differential expression analysis among subgroups with different m^6^A modification patterns based on R package “limma”, which implemented an empirical Bayesian algorithm to identify DEGs (39). We considered genes with adjusted *p* values < 0.05 as statistically different DEGs and utilized these for downstream analysis.

### Collection of Expression Data Matching Immunotherapy Response Information

In order to investigate potential predictive values of m^6^A modifications for immunotherapy response in GBM patients, we comprehensively searched expression data matching anticancer responses for PD-L1 treatment. A urothelial cancer cohort treated with anti-PD-L1 antibody was finally included for downstream analysis (40). The entire expression data and matching PD-L1 response information can be wholly obtained from R package IMvigor210CoreBiologies. For raw expression data in the R package, we adopted the function filterNvoom to normalize and filter out genes with low reads.

### Construction and Validation of MPAP

Using DEGs obtained from subgroups with distinct m^6^A signatures, we aimed to construct a scoring system in order to estimate GBM patients’ prognosis. Firstly, R package rbsurv, a modeling tool to produce numerous Cox models and then select the optimum one, was applied to filter the survival-related genes for the purpose of enhancing the robustness using cross-validation methods. Next, we utilized the least absolute shrinkage and selection operator (LASSO) regression, an efficient regression approach for high-dimensional data with large correlated covariates (41–43), to establish our m^6^A methylation-based immune cell infiltration assessment pipeline (MPAP). Combining Robustness and LASSO regression, we established a MPAP based on 13 genes and its correlation coefficients. Simultaneously, our MPAP was also validated in another GBM cohort. Then, in univariate and multivariate analysis, m^6^A modification scores (MMS) obtained from the MPAP were proven to be independent prognostic factors in both training and verification sets (**Table 1**, **Figure 5**).

**Table 1:** Univariate cox proportional hazards analysis of clinical parameters and m6A risk score level of glioblastoma (GBM) patients in the training set and validation set.

Variables		Training set		Validation set	
		Univariate analysis		Univariate analysis	
		HR(95%CI)	P-value	HR(95%CI)	P-value
Age group	Younger vs old	0.41(0.286-0.61)	**6.7e-06**	1.24(0.87-1.76)	0.217
CIMP status	G-CIMP vs NON	1.63(0.96- 2.78)	0.068	/	/
Gender	Male vs Female	1.04(0.78- 1.40)	0.764	0.91(0.63-1.32)	0.642
Subtype	NE+PE vs CL+ME	1.15(0.80- 1.64)	0.437	/	/
m6Arisk group	Low vs High	0.48(0.34- 0.70)	**8.67e-05**	0.53 (0.32-0.86)	**0.0103**
Radiotherapy	Yes vs No	/	/	0.51(0.26-1.02)	0.05
Chemotherapy	Yes vs No	/	/	0.59(0.30-1.18)	0.139
IDH status	Wildtype vs Mutant	/	/	1.45(0.83-2.53)	0.190
1p19q status	Non vs Codel	/	/	1.20(0.48-2.97)	0.690

Bold values are statistically significant.

### Statistical Analysis

R software (version 3.6.0) was used for all statistical analysis and *p*-values < 0.05 were considered statistically significant. Robustness regression was conducted to select the optimum Cox model and LASSO regression was subsequently performed to construct a predictive model. Thereafter, we utilized the Kaplan-Meier (K-M) approach to establish survival curves and log-rank tests to calculate *p*-values between each group. To find the optimum cut-off value of each dataset, we adopted R package survminer, which examined the efficiency of all potential cut-off points. Applying receiver operating characteristic (ROC) curves, we estimated the specificity and sensitivity of the predictive model, which was implemented using R package pROC. Correlation coefficients among 21 m^6^A regulators were calculated using the Spearman correlation analysis and transformed by -log10. In the training set and validation set, we used multivariate analysis and calculated the hazard ratio (HR) to compare the predictive efficacy between the clinical information and our predictive model.

## Results

### Somatic Mutation Frequency Landscape of 21 m^6^A Methylation Regulators

**Figure 1:**
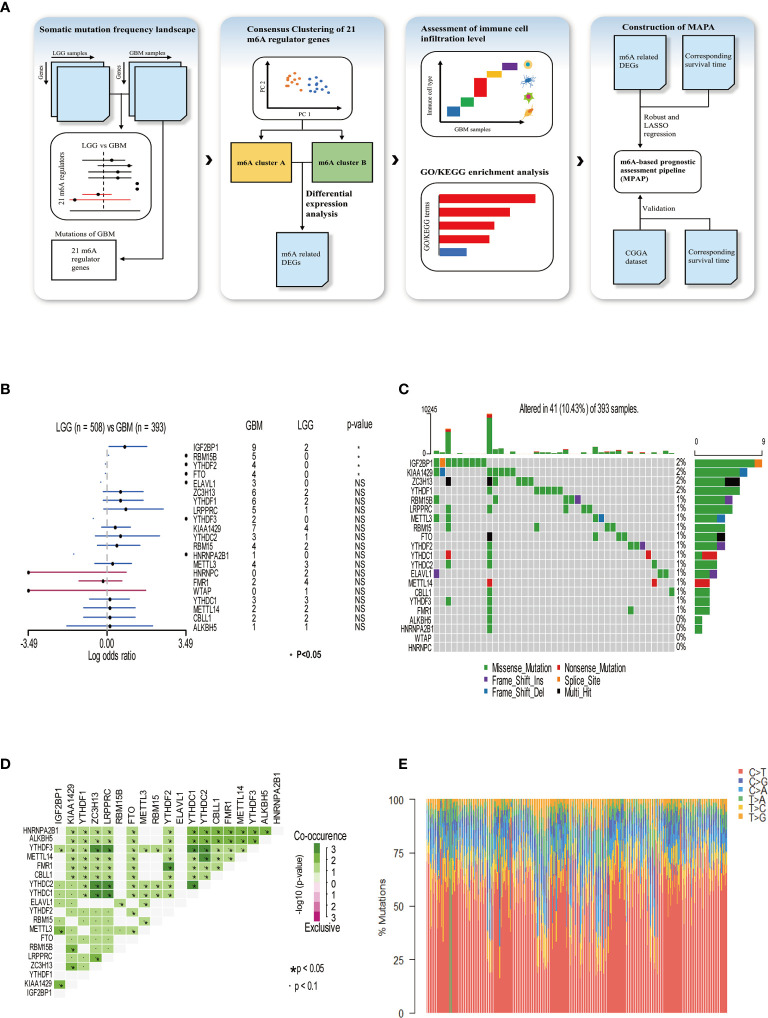
Somatic mutation frequency landscape of 21 m6A methylation modification regulators in GBM. **(A)** Schematic workflow for the construction of MPAP. **(B)** Comparison of somatic mutation frequency of 21 m6A regulators between LGG (n=508) and GBM (n=393). The asterisk means the p-value <0.05. **(C)** The somatic mutation frequency of 21 m6A regulators in 393 GBM patients, in which each column represents a sample. The top bar chart demonstrates tumor mutation burden in each sample. The number on the right shows the somatic mutation frequency of each m6A regulator. The bar chart on the right shows the proportion of mutation types in each regulator. In the middle grid chart, different colors in each lattice represent different types of mutations. **(D)** Correlation of 21 m6A regulators between each other. **(E)** The stacked chart indicates the composition of 393 GBM samples’ base conversion.

In our research, a total of 21 m^6^A RNA methylation regulators was determined, including eight methyltransferases (writer), two demethylases (eraser), and 11 RNA binding proteins (reader). To illustrate the process by which we constructed the MPAP and what datasets were applied in our study, a schematic workflow was developed dividing the overall work into four steps broadly (**Figure 1A**). In **Figure 1B**, we summarized the somatic mutation frequency difference of 21 regulators between low-grade glioma (LGG) and GBM. In 21 regulators, IGF2BP1, RBM15B, YTHDF2, and FTO were significantly higher in GBM than LGG containing one writer (RBM15B), one eraser (FTO), and two readers (YTHDF2, IGF2BP1). Despite the non-significant statistical difference between LGG and GBM among the remaining 17 regulators, the somatic mutation frequency in GBM on m^6^A regulators was considered to be more than in LGG, due to the larger sample size of LGG (508) than GBM (495), except HNRNPC, FMR1, and WTAP, which demonstrated that the somatic mutation frequency of GBM patients among m^6^A regulators tended to be higher than that of LGG patients. This data implies that higher somatic mutation frequency of m^6^A regulators may contribute to the malignant degree of gliomas. The somatic mutation frequency of 21 m^6^A regulators was depicted in GBM samples. Totally, in 495 GBM samples, 41 obtained alterations accounting for 10.43%. IGF2BP1 displayed the highest mutation rate, while WTAP and HNRNPC did not display any mutation (**Figure 1C**). Next, we further demonstrated the co-occurrence of 21 regulators, among which YTHDC1/2 and ZC3H13, YTHDC1/2 and LRPPRC, YTHDC1/2 and YTHDF3, YTHDC1 and YTHDC2, YTHDF3 and ZC3H13, YTHDF3 and LRPPRC, YTHDF2 and FMR1, and YTHDC2 and METTL14 exhibited significant correlation (**Figure 1D**). The composition of 495 GBM samples’ base conversion is shown in **Figure 1E**. Additionally, based on the transcriptome expression level of 21 m^6^A regulators, GBM samples can be entirely discriminated against normal tissue using PCA analysis (**Supplemental Figure 1**).

### Expression Pattern Based on 21 m^6^A Methylation Modification Regulators

**Figure 2:**
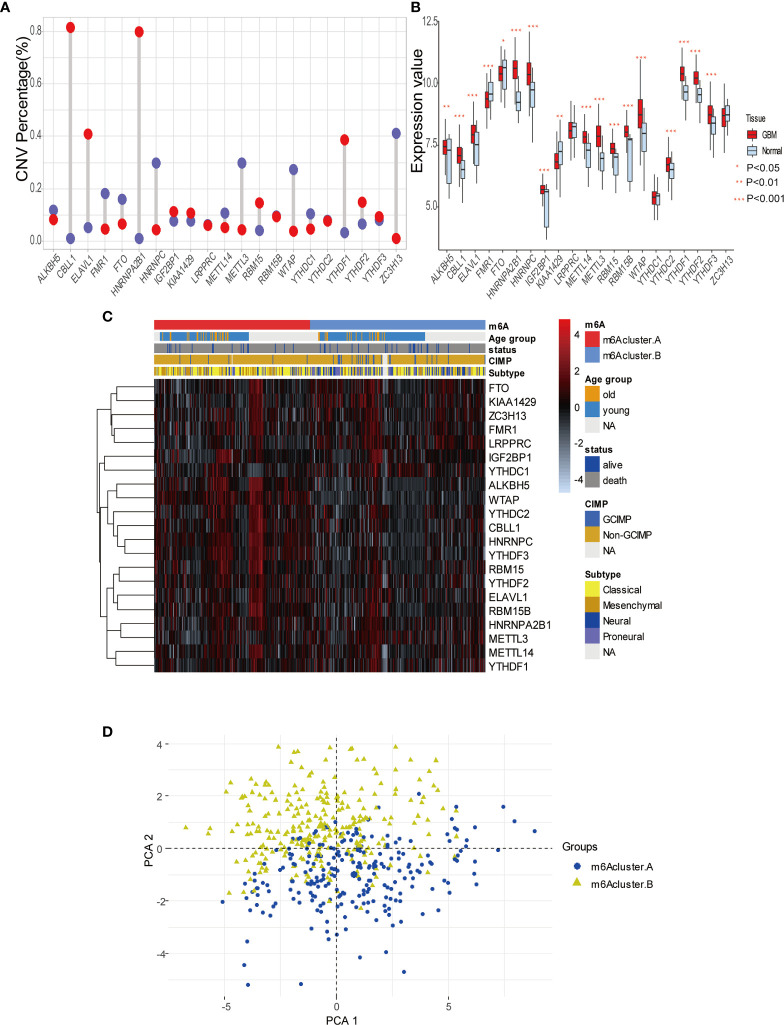
Expression pattern of 21 m^6^A modification regulators in GBM. **(A)** The copy number variation (CNV) percentage of m^6^A regulators in GBM. The red dot represents the CNV amplification and the blue dot represents the CNV deletion. **(B)** The expression value of each m^6^Aregulators between tumor and normal sample. **(C)** The heatmap indicating the expression pattern of m^6^A regulators between 2 m^6^A modification clusters, which matched the clinical information, including age, status, CIMP, and histology subtype. **(D)** Principal component analysis (PCA) of m^6^A regulators to differentiate 2 m^6^A clusters.

**Figure 3:**
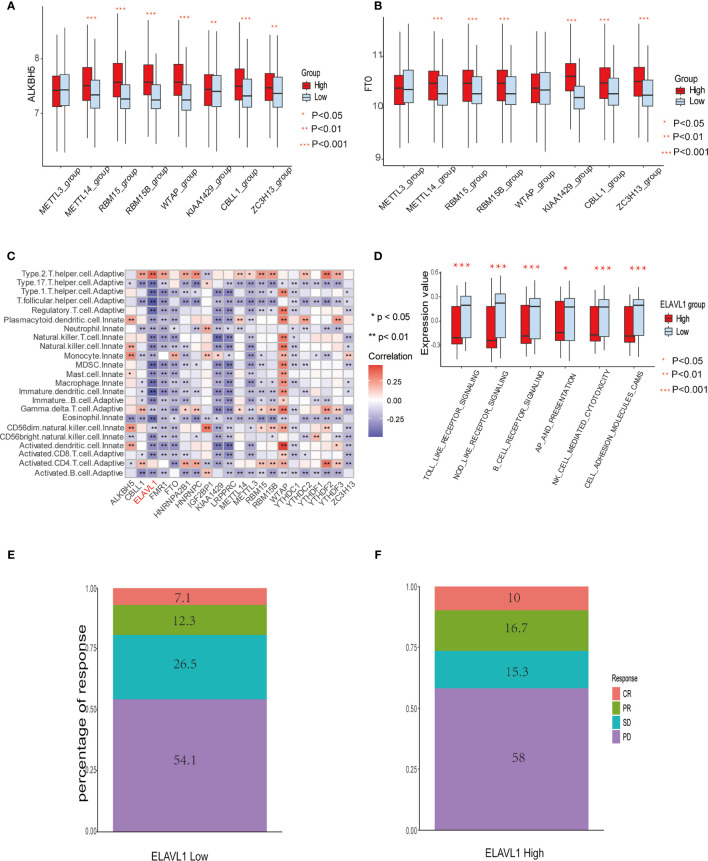
The relationship between m^6^A modification genes and specific immune cell infiltration. **(A)** The expression level of eraser ALKBH5 between high and low expression groups of writers, including METTL3/14, RBM 15/15B, WTAP, KIAA1429, CBLL1, and ZC3H13. **(B)** The same as A in eraser FTO. **(C)** Correlation between 23 immune cells infiltration level and 21 m6A modification regulators. **(D)** The enrichment level of immune related pathways in KEGG between high and low expression groups of ELAVL1. **(E, F)** The response percentage for PD-L1 treatment in high and low expression groups of ELAVL1. SD, stable disease; PD, progressive disease; CR, complete response; PR, partial response.

To explore m^6^A modification patterns in GBM, we included several GEO datasets and matching clinical information for integrative analysis. Applying copy number variation (CNV) alteration analysis, we observed widespread CNV alteration on 21 regulators, among which amplification and deletion vary. CBLL1, HNRNPA2B1, ELAVL1, and YTHDF1 displayed the prevalent CNV amplification, while ZC3H13, HNRNPC, METTL3, and WTAP displayed the opposite (**Figure 2A**). We further analyzed the transcriptome expression level of 21 m^6^A regulators in GBM patients. Results indicate that regulators with CNV amplification tend to exhibit higher mRNA expression levels compared to normal tissue in GBM patients, and vice versa, suggesting that the expression levels of m^6^A regulators are predominantly influenced by CNV alterations (**Figure 2B**). Nevertheless, the transcriptome expression level of some specific regulators including HNRNPC, KIAA1429, METTL14, METTL3, WTAP, is opposite to its CNV alterations. For example, HNRNPC and METTL3 with CNV deletion in GBM tissue have a relatively higher transcriptome expression level than that in tumor tissue. These opposite trends could be attributed to transcriptional events mediated by transcriptional factors and epigenetic changes like histone modifications, DNA methylations, which need to be further elucidated in GBM progression. Adopting R package ConsensusClusterPlus, we divided 495 GBM patients into two clusters with distinct m^6^A modification patterns according to transcriptome expression levels of 21 m^6^A regulators (**Supplemental Figures 2A–C**). In addition, the heatmap of 21 m^6^A regulators, classified by the abovementioned two clusters, demonstrates the relationship between expression level and matching clinical information, including age, clinical status, CIMP, and histology subgroup. Notably, GBM patients in m^6^A cluster B are more likely to express CIMP. Regarding the histology subgroup GBM patients in m^6^A cluster A tend to be in classical and mesenchymal subtype while patients in m^6^A cluster B tend to be in Neural and Proneuronal subtype. And there is no significant difference in the age distribution between two m^6^A clusters (**Figure 2C**). PCA analysis according to transcriptional expression level of m^6^A regulators also completely distinguished between two clusters, implying two m^6^A clusters exist distinct expression profiles of m^6^A regulators (**Figure 2D**). Regulators in the same functional module tend to express similarly. Besides, there is also a significant correlation between methyltransferases and demethylases. For example, among subgroups with higher expression levels of eraser ALKBH5, most writers express the same trend, including METTL14, RBM15, RBM15B, WTAP, CBLL1, and ZC3H13 (**Figure 3A**). Simultaneously, regarding samples with higher expression of FTO (another eraser), we observe a significantly higher level of writers, implying that writers and erasers display a potential interactive effect (**Figure 3B**). In total, 2 m^6^A clusters with potentially different m^6^A modification pattern were determined within the 495 GBM patients based on the transcriptome expression level of 21 m^6^A regulators. m^6^A cluster A was characterized by the relatively low expression of FTO, KIAA1429, ZC3H13, FMR1, LRPPRC, IGF2BP1, YTHDC1 and high expression of the remaining regulators, while m^6^A cluster B showed an opposite trend. Although no significant difference in the age distribution was identified, other corresponding clinical information including CIMP and histology subgroup expressed distinct between 2 m^6^A clusters, indicating that 2 m^6^A clusters with different expression pattern of 21 regulators could have potential mechanisms to mediate these adverse clinical features.

### Immune Cell Infiltration Features in Different m^6^A Modification Modules

Spearman correlation of infiltration levels between 23 immune cells and 21 m^6^A regulators demonstrated that expression level of m^6^Aregulators and TME infiltration closely related to each other. It is suggested that clarifying expression modes of m^6^A regulators is of great significance for forecasting anticancer immune responses, which could be a powerful tool to indicate the efficacy of immunotherapies, such as PD-L1 treatment in GBM patients (**Figure 3C**). We also found that the expression level of the regulator ELAVL1 negatively correlated with the infiltration level of most immune cells, except only type 2 T helper cells and activated CD4+ T cells. Utilizing GSVA to compare immune-related KEGG pathway enrichment degree between subgroups expressing high and low ELAVL1 levels, it was demonstrated that subgroups with high ELAVL1 expression tend to exhibit relatively low enrichment degrees in immune-related pathways and vice versa (**Figure 3D**). Besides, infiltration levels of 23 immune cells and the difference in expression of MHC molecules, costimulatory molecules, and adhesion molecules among subgroups with high and low ELAVL1 show the same trend: the expression of ELAVL1 is negatively correlated to the infiltration level of most immune cells and above-mentioned immune-related modules (**Supplemental Figures 3A, B**).

**Figure 4:**
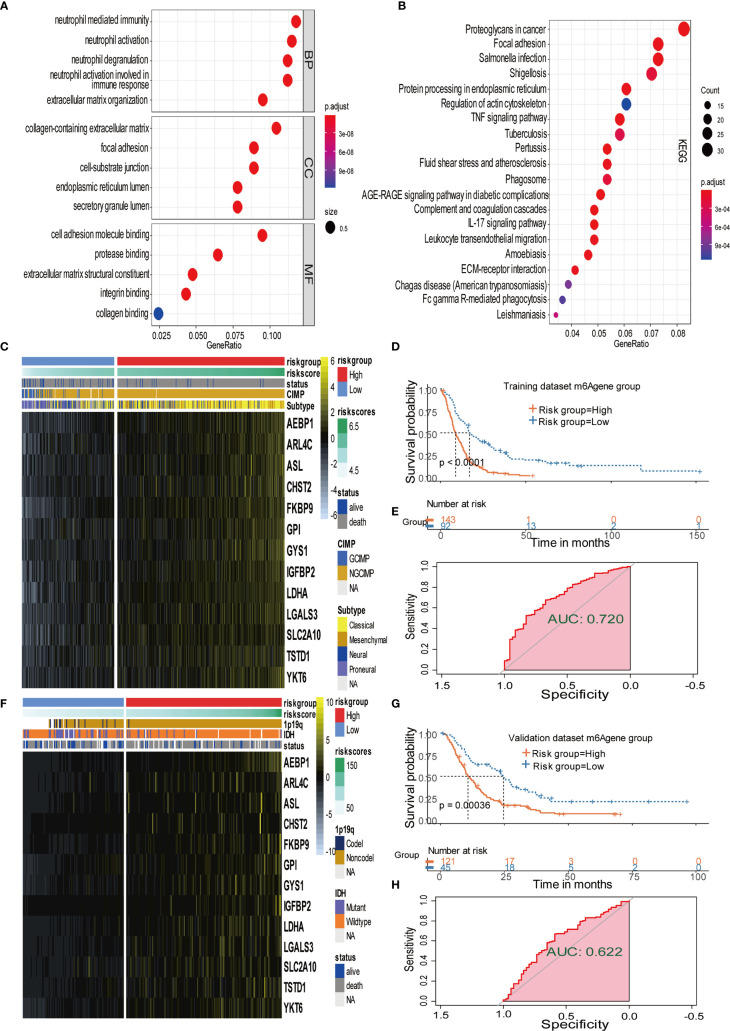
Signature patterns of expression level and biological characteristics of each risk group separated by MPAP. **(A, B)** Bubble plots showing the GO and KEGG annotation of DEGs obtained from two m6A modification clusters. **(C, D) ** Heatmaps demonstrating the expression level of 13 m6^A^A related signatures and the matched clinical information in the training dataset and validation dataset. **(E, F)** Kaplan-Meier survival curves of risk groups separated by MPAP in the training dataset and validation dataset. **(G, H)** ROC curves of the MPAP in the training set and validation set.

The results indicate that m^6^A regulator ELAVL1 is a potential predictive factor of immune response, which could be applied to forecasting the anticancer efficacy of immunotherapy. To elucidate the relationship between expression level of ELAVL1 and response to immunotherapy, we included a urothelial cancer cohort treated with anti-PD-L1 antibody (40). The Kaplan-Meier survival curve between two group classified by ELAVL1 expression did not differ significantly (*p*-value = 0.38, **Supplemental Figure 3C**). In addition, **Figures 3E, F** demonstrate the proportion of patients with response to PD-L1 blockade immunotherapy in low or high ELAVL1 groups, indicating that high ELAVL1 expression correlates with relative efficient responses to PD-L1 treatment. KEGG and GO enrichment analysis for DEGs obtained from two clusters with distinct m^6^A modification patterns indicates that several immune-related KEGG pathways and GO annotation are significantly upregulated, such as neutrophil-mediated immunity and neutrophil activation involved in immune responses, which also supports the results indicating that m^6^A modification patterns are closely correlated to immune response (**Figures 4A, B**).

### Establishment and Validation of m6A Methylation-Based Prognostic Assessment Pipeline (MPAP)

To further explore the potential prognostic value of m^6^A methylation modifications, we constructed the MPAP, which could be used to assess GBM patients’ prognosis. Using DEGs obtained from two clusters with different m^6^A modification patterns for Robustness regression, we chose the optimum Cox modeling gene for the construction of our pipeline. Thereafter, LASSO regression was applied for the establishment of the MPAP, during which 13 genes and the correlation coefficients were obtained (**Supplemental Figures 4A, B**). According to the expression value of 13 genes and the correlation coefficients, we computed the m^6^A modification score to divide GBM patients into groups with distinct clinical outcomes (**Figure 4D**). The overall survival of the high-risk m^6^A modification group is significantly shorter than that of the low-risk group with a log-ranked *p*-value < 0.0001. The expression levels of 13 genes (AEBP1, ARL4C, ASL, CHST2, FKBP9, GPI, GYS1, IGFBP2, LDHA, LGALS3, SLC2A10, TSTD1, YKT6) are depicted in the heatmap (**Figure 4C**).

Simultaneously, for the purpose of testing the robustness of the MPAP, we utilized another GBM cohort from CGGA for prognostic prediction as a validation dataset. Similarly, GBM patients in the validation set were divided into two m^6^A modification pattern-based risk groups according to MMS obtained using the MPAP. The expression levels of 13 genes are also depicted in the heatmap (**Figure 4F**). The Kaplan-Meier curve depicted by matching clinical outcomes reveals a similar trend: the overall survival of the high-risk m^6^A modification group is significantly shorter than that of the low-risk group (log-ranked *p*-value = 0.00036), which confirms the robustness of our model in a different GBM cohort (**Figure 4G**).

**Figure 5:**
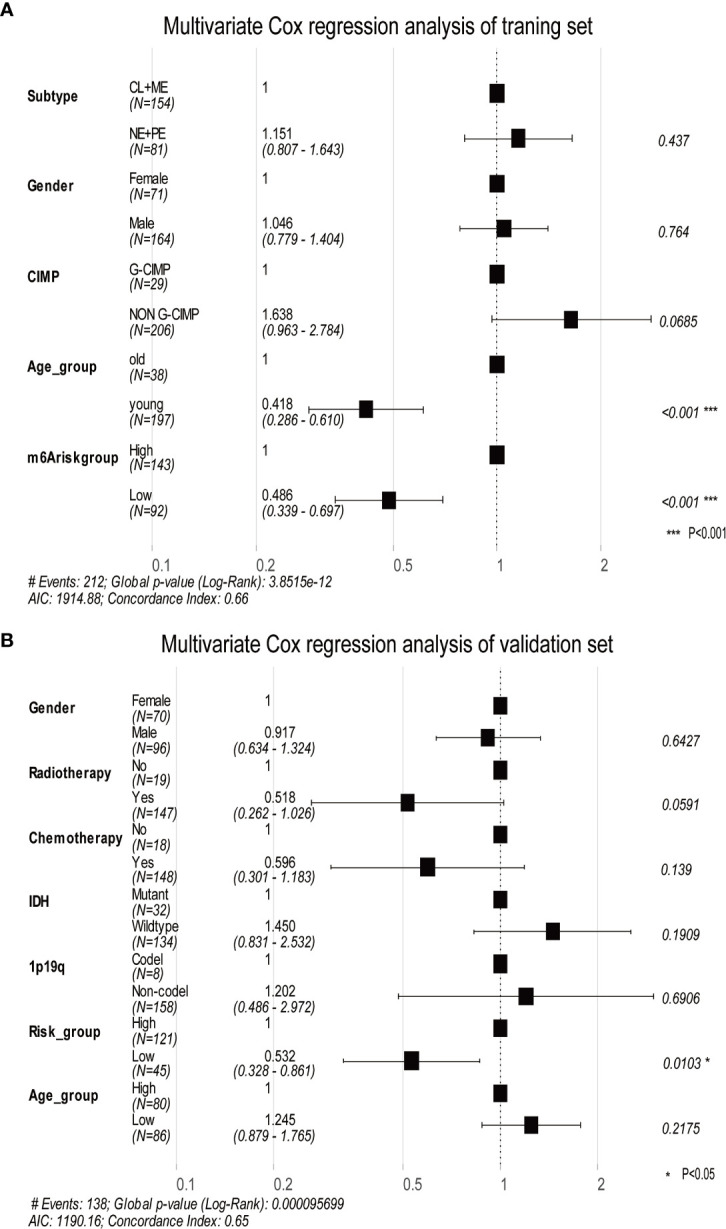
The multivariate Cox regression analysis depicts log-ranked p-values of each factor to predict the prognosis in the training dataset and validation dataset. **(A)** The forest plot shows the results of multivariate Cox regression analysis in the training dataset, in which the black squares represent the Hazard Ratio (HR) and the whiskers around the squares represent the 95% confidence interval. The figures on the left side are the HR of each predictor while on the right side are the p-value. **(B)** The same as A in the validation dataset. AIC, Akaike information criterion.

**Figure 6:**
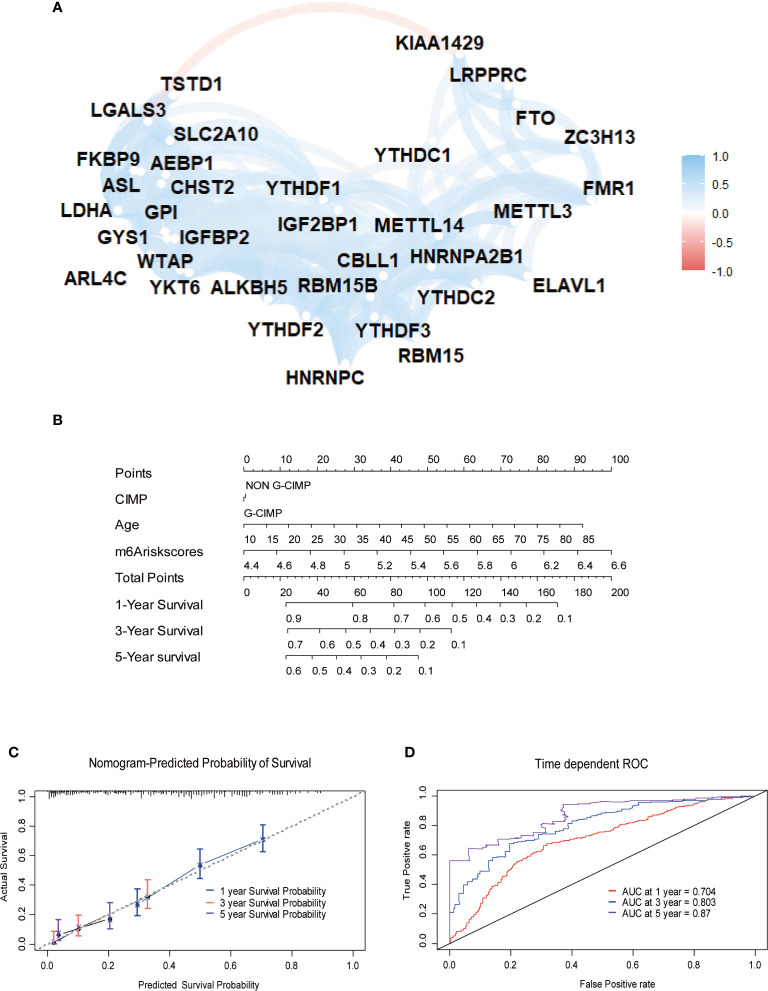
Construction of quantitative prediction nomogram based on methylation-based prognostic assessment pipeline (MPAP). **(A)** The network diagram shows the interaction between 21 m^6^A regulators and 13 m^6^A related signatures. **(B)** A nomogram to quantitatively predict survival based on m6A modification score (MMS), clinical, and molecular parameters. **(C)** Calibration curves of the nomogram demonstrates the accuracy of the predicted survival. **(D)** Receiver operating characteristic (ROC) curves to estimate the performance of the predictive nomogram.

Furthermore, we depicted ROC curves of the predictive model in the training and validation sets. The MPAP displays satisfactory prediction sensitivity and specificity with the area under the ROC curve measuring 0.720 and 0.622 in the training and validation sets, respectively (**Figures 4E, H**). The multivariate Cox regression analysis confirmed that the MPAP was an independent prognostic predictor in GBM patients with a log-ranked *p*-value <0.001 (**Figure 5A**), and the same result was obtained in the validation set (**Figure 5B**). To reveal the correlation between 21 m^6^A regulators and 13 signatures in the MPAP, we depicted a network diagram indicating that most regulators and signatures are regulated positively by each other, except TSTD1 and KIAA1429 (**Figure 6A**). In addition, a nomogram was established to quantitatively forecast the clinical outcomes matching other clinical predictors, which indicates that the MMS was the most valuable predictor (**Figure 6B**). The calibration plot simultaneously demonstrated superior clinical predictive efficiency (**Figure 6C**). In ROC curve, area under the curve (AUC) at 1 year, 3 year and 5 year were 0.704, 0.803 and 0.87 respectively, which indicates that the nomogram have a superior sensitivity and specificity in predicting the probability of survival (**Figure 6D**).

## Discussion

GBM is a group of heterogeneous intracranial neoplasms with distinct histopathological and molecular biological characteristics, resulting in different subsets of patients benefitting from various treatment strategies, despite advancement in multiple therapies for malignant gliomas, including checkpoint inhibitors and targeted therapies et al. (6, 43, 44). To solve this problem, it is urgent to establish a risk stratification method and classify GBM patients into various risk groups with diverse anticancer responses to various therapies. Although the classical approach for identifying specific targets and assessing prognosis has made remarkable achievements, such as immunohistochemistry (IHC) and traditional histopathology, we require more comprehensive and thorough tools to adapt the changing treatment strategies. Despite its powerful efficiency in detecting potential therapeutic targets and eliminating interference of intra-tumoral heterogeneity, scRNA-seq cannot be applied to clinical settings due to the high costs of sequencing and is thus only used for laboratory research. Recently, many studies have suggested that m^6^A methylation modification influences multiple processes during cancer progression, such as inflammation and specific cellular signaling pathways (45). Therefore, applying next generation sequencing (NGS) to explore m^6^A methylation modification patterns in GBM will contribute to the classification of GBM patients for precision medicine and individualized treatment.

Recently, mRNA m^6^A methylation modification was reported to have significant role in multiple immune-related biological process including innate and adaptive immune response, immune cell homeostasis, immune recognition as well as anti-tumor immunity (14, 46–48). Although various evidence suggested single m^6^A regulator is related to individual type of immune cell and specific aspect of immunity (48), integrated analysis of 21 m^6^A regulators to determine its relationship with multiple immune cell infiltration has not been conducted in GBM. Since substantial evidence demonstrated that some mutational signature can be utilized to predict a poor T cell infiltration, low survival rate and multiple systematic therapy resistance in gliomas especially for PD-1 blockade (49), comprehensive recognition of epigenetic modification mediated immune cell infiltration feature in intracranial malignancies can provide novel insights into risk stratification and clinical therapeutic strategy. Hence, we identified 2 diverse m^6^A methylation modification patterns in GBM patients with distinct tumor microenvironment immune cell infiltration utilizing 21 m^6^A modification regulators. However, the concrete mechanism regarding how regulators influence immune cell infiltration and proceed to regulator immune responses requires further clarification. In addition, it is important to elucidate the interaction and mechanism among m^6^A regulators and to find hub regulators that could be adopted for GBM treatment. Our research provides practical ideas for the above-mentioned challenges. However, further research is required to understand how m^6^A modification affects immune response.

Increasing evidence suggested that m^6^A modification play an extensive role in antitumoral drug resistance through various mechanisms including drug transport and metabolism, mutational drug targets, cellular damage repair etc (50). It was also reported that targeting some specific m^6^A regulators can substantially surmount drug resistance in several cancers (51–53). Notably, previous study demonstrated that methylation modification of MGMT promoter can lead to increased chemotherapeutic effect in high grade gliomas (54, 55). Therefore, identifying epigenetic modification related targets will contribute to enhancing anticancer effect of systematic treatment in GBM especially of immunotherapy. And integration of multiple therapy and choosing the optimum treatment strategy on the basis of advanced risk stratification model may be the future direction for treating intracranial malignancies. It was also confirmed that DEGs obtained from differential expression analysis between two clusters with different m^6^A methylation modification modes were closely related to epigenetic and immune response-related KEGG pathways and GO terms, for example, neutrophil-mediated immunity and neutrophil activation involved in immune responses. We considered these m^6^A modification related DEGs as key signature genes in GBM, which could be utilized to detect potential characteristics within intra-tumoral heterogeneity of GBM, including immune cell infiltration. Thus, using these m^6^A modification-related signature genes, the MPAP was constructed to quantitatively assess m^6^A patterns in GBM. Hence the MPAP could be used as a novel tool to relatively assess GBM patients’ prognosis and guide clinical decision-making. As the means of GBM therapy diversifies, formulating treatment strategy based on prognostic models to guide precision medicine will be a trend in the fields of cancer treatment (56, 57).

However, our study has a few limitations: retrospective research displays statistical bias and the traditional bulk sequence transcriptome data lack comprehensive exploration for the intra-tumoral heterogeneity. Admittedly, there is urgent demand for a prospective study to acquire a superior fit. Yet, we have established a superior predictive model to quantitatively assess GBM patients’ clinical outcomes based on m^6^A modifications through multiple transcriptome data, at a relatively low price that could be widely used.

Additionally, although recent study had explored the connection between tumor mutational burden and immunotherapy response in gliomas (49), the influence of specific gene for the efficacy of immunotherapy in GBM has not been elucidated due to the small sample capacity in the research on the effect of immunotherapy for GBM. Instead, we applied a urothelial cancer cohort treated by PD-L1 blockade to explore the connection between transcriptome expression pattern and immunotherapy response and ELAVL1 was determined to be a predictor for the efficacy of PD-L1 blockade. But that urothelial cancer cohort is different from GBM in some respects, for example, tumor samples in that cohort are metastases. And urothelial cancer is thought to be in a totally different immune subtype from GBM (58), which will affect the prediction value of ELAVL1 on immunotherapy. To clarify these confusions, we further explore the correlation between the expression level of 21 m6A regulators and immune cells infiltration level in that urothelial cancer cohort (**Supplemental Figure 5**). It was demonstrated that the correlation between ELAVL1 and 23 immune cells infiltration level in the urothelial cancer has a similar trend with that in GBM cohort (**Supplemental Figure 5**, **Figure 3C**), which support our conclusion to a certain extent. However, due to the heterogeneity between urothelial cancer and GBM the prediction value of ELAVL1 and its potential mechanism for immunotherapy still needs to be further proved by large glioma cohort treated by PD-1/L1 blockade.

To summarize, comprehensively studying the m^6^A methylation modification patterns in GBM patients, two diverse m^6^A phenotypes with distinct epigenetic modification modes were identified to explore m^6^A modification-related signatures. Using a quantitative method to assess the infiltration level of 23 immune cells in transcriptome expression data, we integrated these m^6^A regulated signature genes for further analysis to determine the relationship between immune responses and m^6^A modifications, which we could apply to estimate anticancer responses to immunotherapies in clinical practice. According to our results, m^6^A modification regulator ELAVL1 was identified to potentially play a role in the efficient prediction of PD-L1 treatment, while the effect of other m^6^A regulators on specific treatment strategies were to be determined. Considering the urgent demand for the construction of a risk stratification and prognosis assessment system in GBM patients to cautiously formulate treatment management, we established the MPAP using the m^6^A-related signature genes to assess the m^6^A modification level, by which immune cell infiltration level can be identified and then be used to predict the clinical outcomes for patients receiving immunotherapy treatment. Furthermore, integrating other clinical information, including CIMP and age, we constructed a nomogram to precisely forecast GBM patients’ survival time, in which the MMS obtained from MPAP was the leading predictor. In short, our findings provided a comprehensive understanding of m^6^A modifications in GBM and provided a powerful, high quality tool at a low cost to quantitatively estimate GBM patients’ therapeutic response and clinical prognosis.

## Conclusions

In conclusion, by detecting distinct expression patterns of 21 m^6^A modification regulators in GBM, this study successfully identified 13 m^6^A-related signatures and constructed the MPAP combining the Robust and LASSO regression, which we could employ to quantitively predict the prognosis of GBM patients. Additionally, we also determined that m^6^A regulators are correlated with specific immune cell infiltration levels. Comprehensively exploring m^6^A modification patterns in GBM will enhance our understanding of immune infiltration features in order to better manage the treatment strategies and improve clinical outcomes.

## Data Availability Statement 

Publicly available datasets were analyzed in this study. These data can be found here: https://xena.ucsc.edu https://gliovis.bioinfo.cnio.es.

## Ethics Statement

Written informed consent was obtained from the individual(s) for the publication of any potentially identifiable images or data included in this article.

## Author Contributions

YP and KX wrote the manuscript and drew the figures. YL and YZL collected the data. QL wrote the manuscript and supervised the entire project. All authors contributed to the article and approved the submitted version.

## Funding

This work was supported by the National Natural Science Foundation of China (grant number 81802974)

## Conflict of Interest

The authors declare that the research was conducted in the absence of any commercial or financial relationships that could be construed as a potential conflict of interest.

## Supplementary Material

Supplementary Figure 1 Principle component analysis (PCA) of m6A modification genes to differentiate normal and tumor tissue.

Supplementary Figure 2 Consensus clustering of GBM patients’ m6A modification regulators. **(A)** Consensus matrices of the GBM cohort for k (number of clusters) = 2 dividing GBM patients into two clusters. **(B)** Empirical cumulative distribution function (CDF) plot display consensus distributions for each k, which suggests dividing the patients into 2 groups reach the maximum stability. **(C)** The relative change area under CDF curve (y-axis) indicates the relative increase in cluster stability, which means the optimal k=2.

Supplementary Figure 3
**(A)** infiltration level of 23 immune cells between groups with high and low ELAVL1. **(B)** Expression level of MHC molecules, co-stimulatory molecules, and adhesion molecule between groups with high and low ELAVL1. **(C)** Kaplan-Meier survival curves of groups with high and low ELAVL1 expression level.

Supplementary Figure 4
**(A)** LASSO coefficient profiles of the m6A related signatures. **(B)** Using 10-fold cross-validation to the optimal penalty parameter lambda.

Supplementary Figure 5 Correlation between 23 immune cells infiltration level and 21 m6A modification regulators.
