# Supplementary figures and images for: RNA N6-Methyladenosine Regulator-Mediated Methylation Modifications Pattern and Immune Infiltration Features in Glioblastoma
Source: Front Oncol. 2021 Feb 25;11:632934. doi: 10.3389/fonc.2021.632934 (PMC7947873; doi:10.3389/fonc.2021.632934)

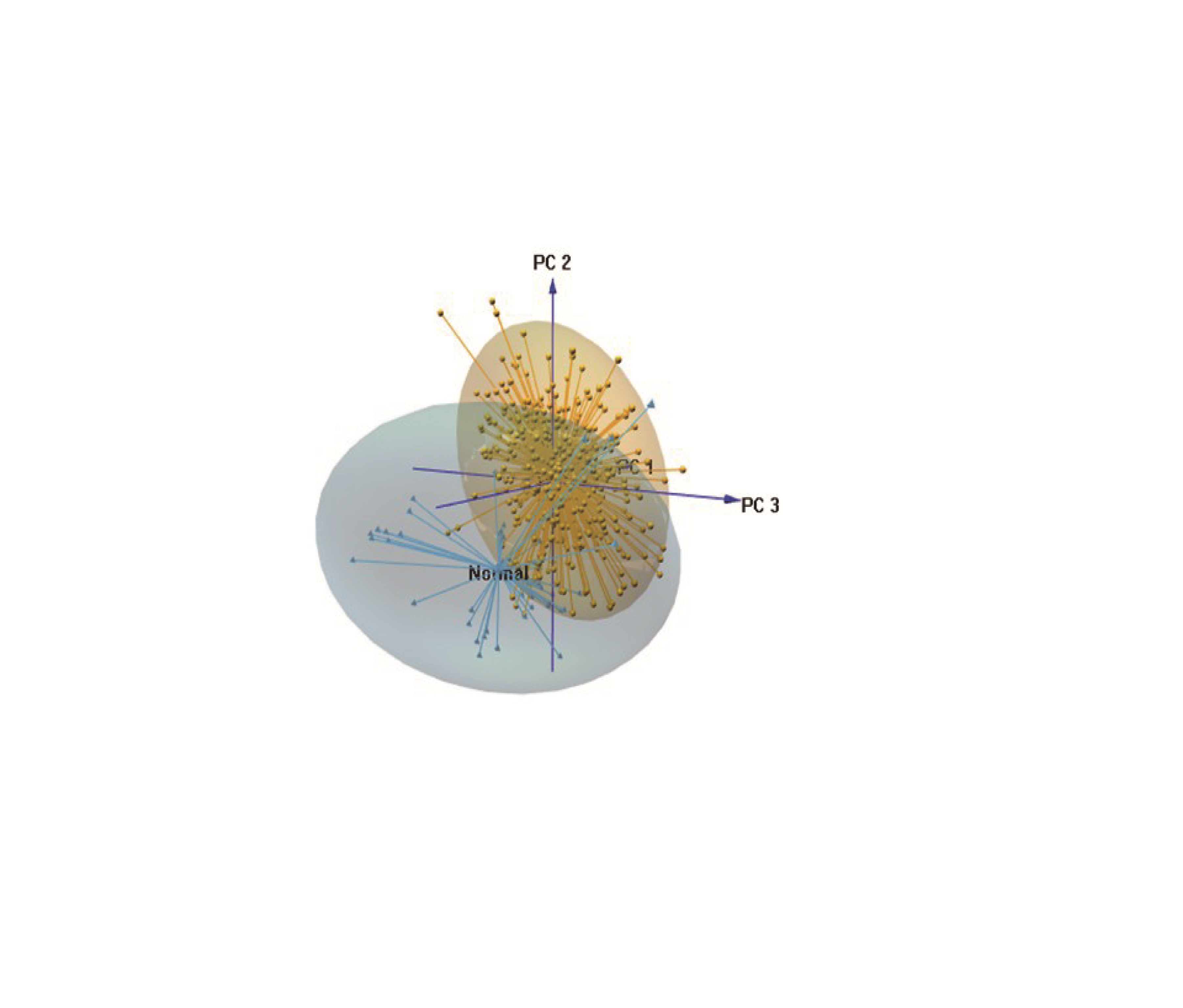

Supplement: Supplementary Figure 1 — Principle component analysis (PCA) of m6A modification genes to differentiate normal and tumor tissue. [file Image_1.tif]

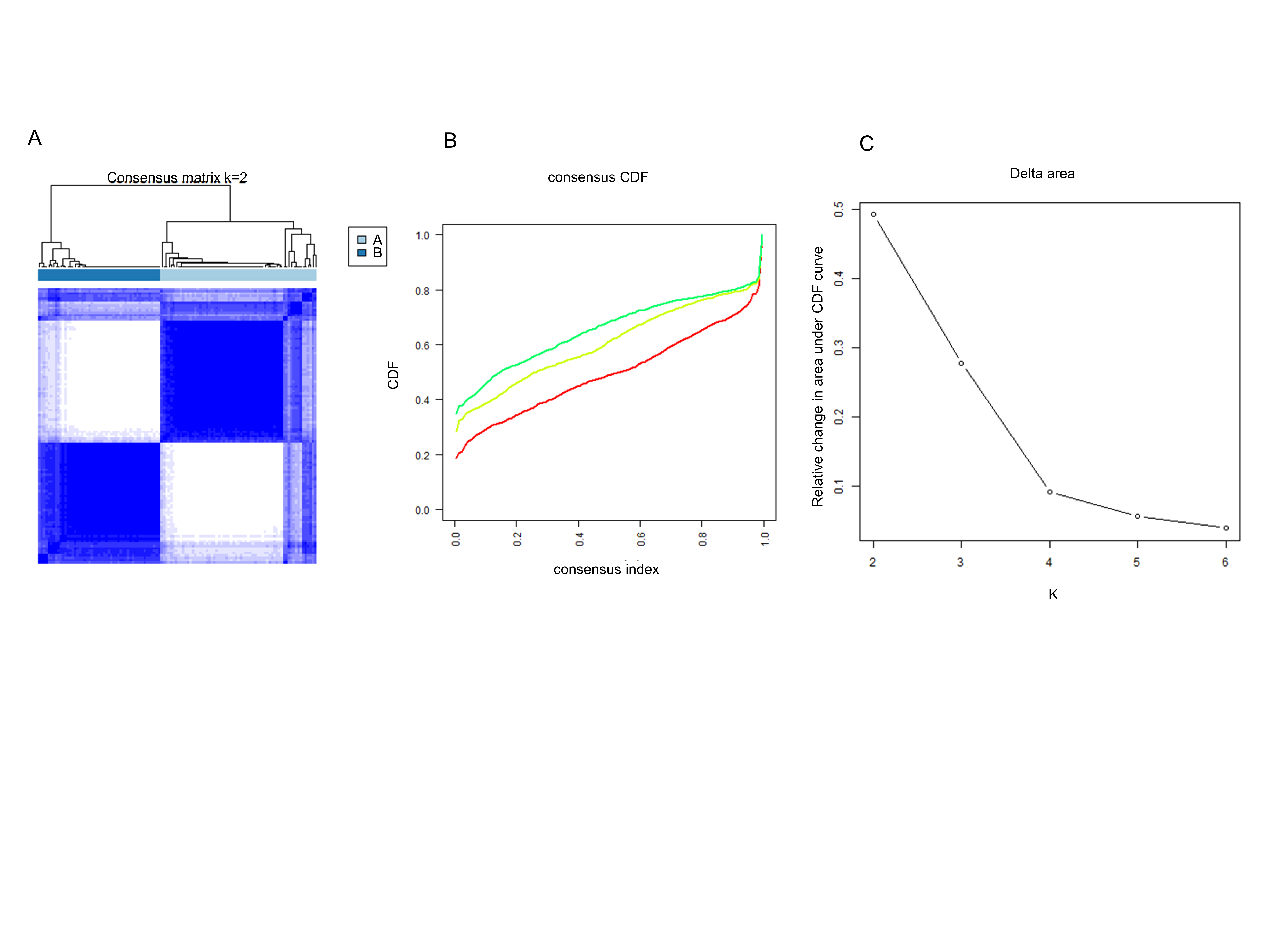

Supplement: Supplementary Figure 2 — Consensus clustering of GBM patients’ m6A modification regulators. (A) Consensus matrices of the GBM cohort for k (number of clusters) = 2 dividing GBM patients into two clusters. (B) Empirical cumulative distribution function (CDF) plot display consensus distributions for each k, which suggests dividing the patients into 2 groups reach the maximum stability. (C) The relative change area under CDF curve (y-axis) indicates the relative increase in cluster stability, which means the optimal k=2. [file Image_2.tif]

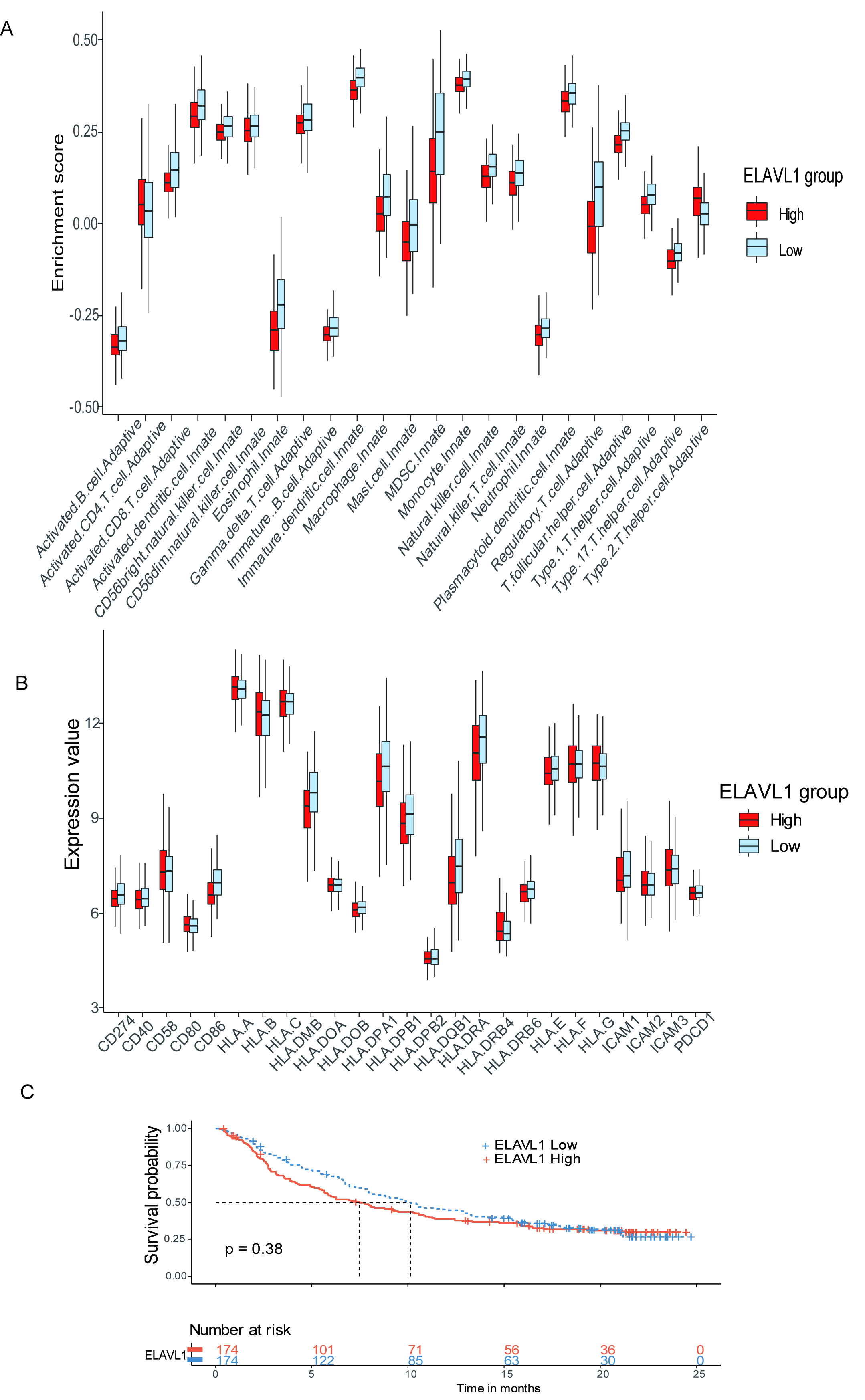

Supplement: Supplementary Figure 3 — (A) infiltration level of 23 immune cells between groups with high and low ELAVL1. (B) Expression level of MHC molecules, co-stimulatory molecules, and adhesion molecule between groups with high and low ELAVL1. (C) Kaplan-Meier survival curves of groups with high and low ELAVL1 expression level. [file Image_3.tif]

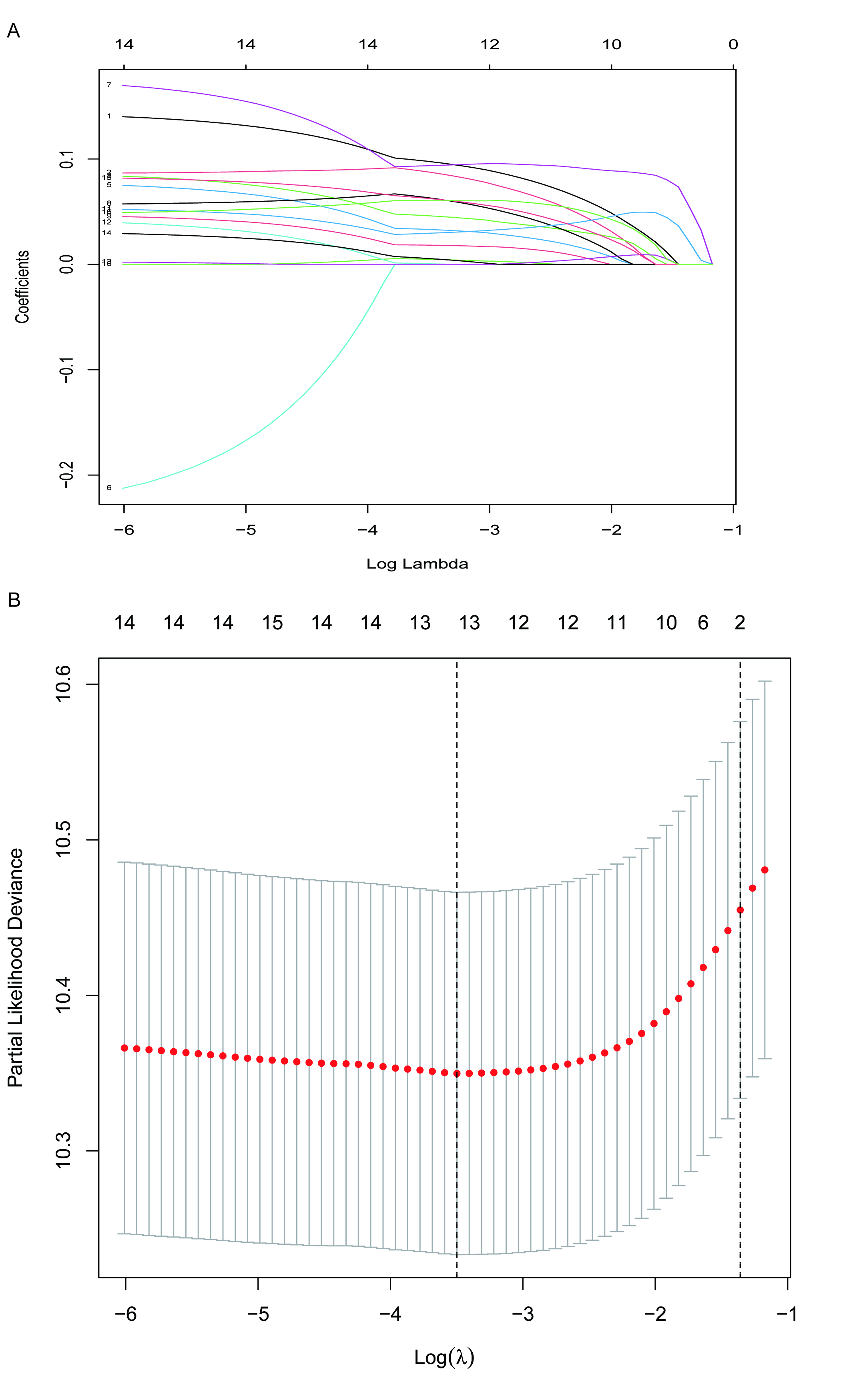

Supplement: Supplementary Figure 4 — (A) LASSO coefficient profiles of the m6A related signatures. (B) Using 10-fold cross-validation to the optimal penalty parameter lambda. [file Image_4.tif]

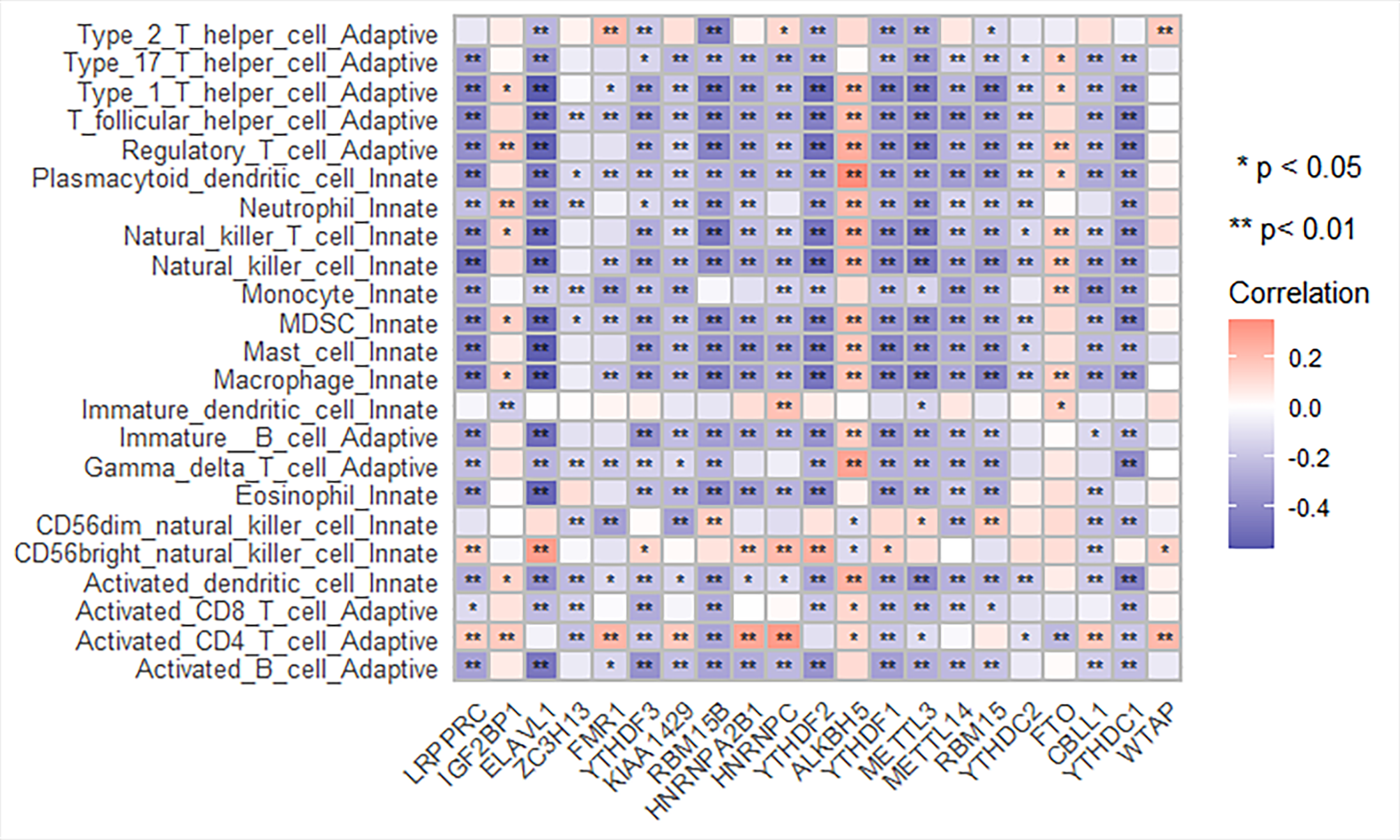

Supplement: Supplementary Figure 5 — Correlation between 23 immune cells infiltration level and 21 m6A modification regulators. [file Image_5.tiff]
